# Simulated Microgravity Impairs Human NK Cell Cytotoxic Activity Against Space Radiation-Relevant Leukemic Cells

**DOI:** 10.21203/rs.3.rs-3972868/v1

**Published:** 2024-04-30

**Authors:** Christopher Porada, Bradford Kuhlman, Jonathan Diaz, Trang Simon, Kimberly Reaves, Stephen Walker, Anthony Atala, Graça Almeida-Porada

## Abstract

Natural killer (NK) cells are important effectors of the innate immune system. Unlike T cells, NK cells do not require antigen-priming, making them an important first-line of defense against malignant cells. Because of the potential for increased cancer risk as a result of astronaut exposure to space radiation, we performed studies to determine whether conditions of microgravity present during spaceflight affects the body’s natural defenses against leukemogenesis. Human NK cells were cultured for 48 hours under normal gravity and simulated microgravity (sµG), and cytotoxicity against K-562 (CML) and MOLT-4 (T-ALL) cell lines was measured using standard methodology or under continuous conditions of sµG. Even this brief exposure to sµG markedly reduced NK cytotoxicity against both leukemic cells using standard assay procedures, and these deleterious effects were even more pronounced in continuous sµG. RNA-seq performed on NK cells from two healthy donors provided insight into the mechanism(s) by which sµG reduced cytotoxicity. Given our prior report that human HSC exposed to simulated space radiation gave rise to T-ALL *in vivo* , the reduced cytotoxicity against MOLT-4 is striking and raises the possibility that µG may add to astronaut risk of leukemogenesis during prolonged missions beyond LEO.

## INTRODUCTION

Natural killer (NK) cells are effector lymphocytes of the innate immune system that perform immunosurveillance and provide a first line of defense against virally-infected cells and cells that undergo malignant transformation, recognizing them and eliminating these aberrant clones, without the need for prior sensitization, before they can proliferate and give rise to disease [1]. In addition to mediating direct cytolysis of tumor cells, NK cells also serve as a critical bridge to the adoptive immune system, releasing cytokines such as IFN-γ, TNF-α, GM-CSF, and IL-33 and the chemokines MIP-1α, MIP-1β, and RANTES to recruit circulating T cells to the tumor site and stimulate T cell activation and proliferation to enhance the body's immune response against the cancer [2,3]. As such, any stressors that negatively affect the inherent anti-tumor properties of NK cells could directly increase the risk of carcinogenesis. During future missions beyond low Earth orbit (LEO), such as those NASA and other space agencies are planning to the Moon, near-Earth asteroids, and Mars, astronauts will be exposed to a complex ionizing radiation (IR) environment unlike anything present on Earth, consisting of solar energetic particles (SEP) and galactic cosmic rays (GCR) that contain high atomic number and energy (HZE) charged particles. The high linear energy transfer (LET) and correspondingly higher relative biological effectiveness (RBE) of HZE particles will expose astronauts on long-duration missions to cumulative IR doses that could increase cancer risk [4–7]. This possibility led NASA to identify space radiation-induced carcinogenesis as one of the top "red" risks limiting long-duration missions in deep space [8,9] [https://humanresearchroadmap.nasa.gov/intro/*].* Highlighting the potential for carcinogenesis as a result of exposure to the types and doses of IR to which astronauts will be exposed when they ultimately venture beyond LEO, we reported [10] the induction of human T cell acute lymphoblastic leukemia (T-ALL) in mice that were repopulated with human hematopoietic stem cells (HSC) that had been exposed to Mars mission-equivalent doses [11,12] of $^{56}Fe$ ions. While these findings are concerning in their own right, the well documented dysregulation of multiple facets of the immune response that occurs during even short duration spaceflight [13–16] suggests that accurate cancer risk assessment during future multiple-year missions in deep space will require consideration of not just the incidence of carcinogenesis arising from IR exposure, but also the fitness of the astronauts' anti-tumor immunity.

Given their key role in anti-tumor immunity, several studies have begun addressing the effect of spaceflight on the functionality of human NK cells [17–25]. While these studies have collectively revealed that spaceflight stressors, in particular microgravity, negatively impact NK cells, it is important to note that these studies were all conducted on Earth under conditions of normal gravity. Specifically, in the studies utilizing NK cells from crew members, cells were isolated during or immediately following spaceflight, and they were then cocultured with target cells under normal gravity. Similarly, in all of the *in vitro* studies using ground-based analogs of microgravity such as the NASA-designed Rotating Wall Vessel (RWV - Synthecon, Inc., Houston, TX, USA), cytotoxicity assays were performed after culture of NK cells for a period of time in simulated microgravity, followed by centrifugation and subsequent coculture of NK cells with target cells under conditions of normal gravity for a period of 4 hours. Since it is well documented that dramatic changes in gene expression occur within seconds in response to changes in gravity [26–29], it is safe to posit that the hypergravity experienced during centrifugation as well as the 4-hour incubation period with target cells under conditions of normal gravity likely prevented a truly accurate assessment of NK function in "real-time" during spaceflight.

To better model the ability of astronauts' NK cells to continually monitor for and eliminate any transformed cells that emerge as a result of exposure to SEP/GCR radiation, and thereby be able to accurately estimate cancer risk, we performed studies, using a modified workflow that enabled us to maintain both the NK cells and the target tumor cells in a continuous state of microgravity throughout the entire assay period, to more accurately evaluate NK cell tumor surveillance and elimination during spaceflight. We utilized both standard K562 cells and T-ALL cells as targets, given our prior work demonstrating the induction of this malignancy as a result of exposure of human HSC to Mars mission-equivalent doses of GCR ions. Results obtained in conditions of continuous microgravity were then compared to those obtained with "traditional" methods in which NK cells were exposed to simulated microgravity, centrifuged, and then cocultured with target cells under normal gravity. Our results show that NK function is significantly more impaired when simulated microgravity conditions are maintained uninterrupted throughout the cytotoxicity assay period, suggesting prior work may have underestimated the impact microgravity exerts on these critical immune cells. Of particular note was the marked reduction in cytotoxicity against T-ALL, raising the troubling possibility that the combined exposure to space radiation and conditions of microgravity may place astronauts at even greater risk of this malignancy during prolonged missions beyond LEO. RNA-seq analyses on NK cells isolated from three healthy adult human donors revealed myriad pathways that were altered by simulated microgravity, paving the way for future studies to develop targeted countermeasures to help mitigate this risk and safeguard astronaut health.

## MATERIALS AND METHODS

### Cell Lines and Tissue Culture

Human natural killer cell line NK-92MI (ATCC® CRL™-2408) was used for all cytotoxicity experiments. During expansion, cells were cultured in standard tissue culture flasks in Alpha Minimum Essential medium without ribonucleosides and deoxyribonucleosides but with 2 mM L-glutamine and 1.5 g/L sodium bicarbonate. Additional components included: 0.2 mM inositol; 0.1 mM 2mercaptoethanol; 0.02 mM folic acid; horse serum to a final concentration of 12.5%; fetal bovine serum to a final concentration of 12.5% (This medium formulation will be referred to as “NK medium” from here forward). T-ALL cell line MOLT4 (ATCC® CRL1582™) and Erythroleukemic cell line K562 (ATCC® CCL243™) were used as target cells. All cell lines used for the present studies were tested and confirmed to be mycoplasma-negative using the MycoStrip™ Mycoplasma detection kit according to the manufacturer’s instructions (InvivoGen US, San Diego, CA, USA). During culture and expansion, these cells were grown in RPMI-1640 + 10% FBS and IMDM + 10% FBS respectively. All cytotoxicity experiments, however, were conducted in complete NK medium, regardless of cell type.

### Microgravity Incubation and Standard Cytotoxicity Assay

NK-92MI cells were cultured in simulated microgravity using Synthecon’s Rotating Cell Culture System (RCCS) in conjunction with High Aspect Ratio Vessels (HARVs). Initially, NK cells were cultured in microgravity for a period of 48 hours. These cells were then removed, centrifuged and resuspended in fresh medium and plated against 500,000 target cells at various effector to target (E:T) ratios. NK and leukemic cell lines were cocultured for a period of 4 hours. The supernatants were then harvested, and cytotoxicity was measured using Promega’s CytoTox 96® Non-Radioactive Cytotoxicity Assay, which measures release of Lactate Dehydrogenase (LDH). Controls accounting for spontaneous release of both target and effector cells, as well as media background were also performed. Cytotoxicity was measured as a percentage of absorbance after complete lysis of target cells (achieved with the addition of 2% Triton X-100).

### Cytotoxicity Assay in Continuous Microgravity

To conduct an assay in which NK cells were continuously exposed to conditions of microgravity, four HARVs were used simultaneously. NK-92MI cells were cultured in simulated microgravity in two HARVs for 48 hours. Without stopping rotation (and therefore maintaining the cells in freefall), the HARVs were sampled to assess cell density and viability. Leukemic cells were then added to one of the HARVs containing NK cells at an E:T ratio of 10:1. Four separate HARVs were implemented containing the following conditions. **A)** NK cells and leukemic cells; **B)** NK cells alone; **C)** leukemic cells alone; **D)** leukemic cells with the addition of 2% Triton X-100. Together, these four vessels were used to determine the cytotoxicity of NK cells against target cells as well as the spontaneous death of NK and leukemic cells individually. These values were then compared to the total lysis of target cells in **D)**. Extreme consideration was taken in order to ensure all cell concentrations and media were identical.

All experiments were also conducted in both standard tissue culture flasks and stationery HARVs in normal gravity to account for any differences in materials. Initial experiments were attempted using HARVs rotating horizontally to account for vibrations and shear stress caused by the motion of rotation, but viability of NK cells after 48 hours of culture in this fashion was so low it prevented cytotoxicity assays from being performed. Additionally, the rationale of using this condition as a control is questionable given that the shear stress experienced by these cells is significantly higher than those in freefall during vertical rotation. Lastly, horizontal rotation results in a centrifugal force vector, causing the cells within the vessel to migrate to the edges. Effectively, this phenomenon would artificially increase cell density at these locations, mostly likely resulting in inaccurate measurements of cytotoxicity.

## RNA Sequencing

RNA was isolated from each cell population using the RNeasy plus kit according to the manufacturer's instructions (Qiagen). cDNA libraries were then prepared from 50 ng of each RNA extract using a NEXTFLEX® Combo-Seq™ mRNA/miRNA Kit (PerkinElmer®, Waltham, MA). Libraries were quantified using a KAPA Library Quantification Kit (Roche Sequencing and Life Science, Indianapolis, IN), with average fragment length determined by a 4200 TapeStation System (Agilent). Libraries were normalized prior to pooling, and the pooled libraries were sequenced using an Illumina® NovaSeq 6000 System (Illumina®, Inc., San Diego, CA), generating 76-bp single-end reads.

## Sequence Analysis

Raw sequence reads were imported into Partek® Flow® software (Partek®, St. Louis, MO) for analysis. Cutadapt[30] was used to trim the random barcode (4 bases) from the 5' end and adapter (AAAAAAAAAA) from the 3' end of the reads. Bases with a Phred quality score < 20 were trimmed, and reads < 15 bases in length were discarded. High-quality reads were then aligned and quantified to the hg38 GENCODE reference database using STAR [31] and an expectation/maximization (E/M) algorithm similar to the one in Xing *et al.* [32], respectively. Transcript-level counts were summed to gene level, normalized with the median-of-ratios method used in DESeq2 [33] and $log_2$-transformed. Principal component analysis (PCA) was used to assess global patterns of expression across exposures.

## RNA-seq Data Analysis

Differential gene expression analysis was performed using ROSALIND® (https://rosalind.bio/), with a HyperScale architecture developed by ROSALIND, Inc. (San Diego, CA). The Benjamini-Hochberg adjusted p-value ($P_{adj}$)[34] and $log_2$ fold change (LFC) was calculated for each gene. A $P_{adj}$ cutoff of 1e-10 was used to eliminate background in each analysis, and the entire gene dataset was used as the reference set for analysis to control for bias. Read Distribution percentages, violin plots, identity heatmaps, and sample MDS plots were generated as part of the QC step. The limma R library[35] was used to calculate fold changes and p-values and perform optional covariate correction. Clustering of genes for the final heatmap of differentially expressed genes was done using the PAM (Partitioning Around Medoids) method using the fpc R library[36] that takes into consideration the direction and type of all signals on a pathway, the position, role and type of every gene, etc. Hypergeometric distribution was used to analyze the enrichment of pathways, gene ontology, domain structure, and other ontologies. The topGO R library[37,] was used to determine local similarities and dependencies between GO terms in order to perform Elim pruning correction. Several database sources were referenced for enrichment analysis, including Interpro[38], NCBI[39], MSigDB[40,41], REACTOME[42], and WikiPathways[43]. Enrichment was calculated relative to a set of background genes relevant for the experiment.

### RNA Sequencing Data Availability

The transcriptomic dataset generated for this study is available in NCBI's Gene Expression Omnibus (GEO) and is accessible through GEO Series Accession Number 255750.

### Statistical Methods

All experiments detailed herein were repeated a minimum of three times to ensure rigor and reproducibility of the data. Data in all figures are presented as the mean ± SEM, and were analyzed with Prism 8 (GraphPad Software, Inc., La Jolla, CA). Statistical significance of differences observed between the various experimental conditions was determined using one-way analysis of variance (ANOVA) followed by the Bonferroni-Šidák correction for multiple comparisons. For all analyses, $p \leq 0.05$ was considered to be statistically significant. Statistical significance is indicated in Figures with * for $p \leq 0.05$ and ** for $p \leq 0.01$.

## RESULTS AND DISCUSSION

### Effect of Microgravity Exposure on the Cytotoxicity of NK-92MI Cells

To begin to define the impact that exposure to microgravity has upon NK cell function, we incubated NK-92MI cells for 48 hours in either: 1) conditions of normal gravity (1G) in a standard culture T-flask; or 2) conditions of simulated microgravity (sµG) using the NASA-designed Synthecon rotating wall vessel (RWV) system and its associated high-aspect ratio vessels (HARVs). At the end of the 48-hour incubation, NK-92MI cells were harvested and their cytotoxicity against two separate leukemic cell lines (K562 – erythroleukemia; MOLT-4 – T-ALL) was measured using a commercially available non-radioactive cytotoxicity assay kit (), following the manufacturer's instructions which involved centrifugation and a 4-hour co-incubation of the NK-92MI cells and leukemic cell lines under 1G. As seen in **Fig. 1**, even a relatively brief (48 hours) exposure of NK-92MI cells to simulated microgravity (sµG) dramatically reduced their ability to kill the erythroleukemic cell line K-562 (Fig. 1A) at all measured E:T ratios, and it significantly reduced their killing of the T-ALL cell line MOLT-4 (Fig. 1B) at higher E:T ratios. A 48-hour exposure of NK-92MI cells to sµG also reduced their killing of MOLT-4 cells at the lowest measured E:T ratio of 5:1, but significance was not achieved.

## Effect of Microgravity Exposure on the Cytotoxicity of Primary Human NK Cells

While the NK-92MI cell line is a workhorse of the NK field, and it has been used in countless studies related to NK function and NK cytotoxicity against various tumors, it is an IL-2-independent permanent cell line, and as such its biology likely differs, at least in subtle ways, from that of primary NK cells. We, therefore, next performed identical studies using primary NK cells isolated from the peripheral blood of three different healthy adult human donors to assess whether a 48-hour exposure to sμG would also impair the cytotoxic function of primary human NK cells. As can be seen in **Fig. 2**, the cytotoxicity of primary NK cells from all (n = 3) healthy donors was also significantly reduced against K562 (Fig. 2A, $p \leq 0.01$) and MOLT-4 (Fig. 2B, $p \leq 0.05$) leukemic cell lines (E:T ratio = 10:1) following a 48-hour exposure to sμG, although the reduction in cytotoxicity was more pronounced against the K562 line.

## Effect of Continuous Microgravity on the Cytotoxicity of Primary Human NK Cells

For the initial studies on NKL-92MI and primary human NK cells, we followed a standard protocol for measuring cytotoxicity in which NK cells were exposed to sμG for 48 hours, followed by centrifugation and subsequent coculture of NK cells with target cells under conditions of normal gravity for a period of 4 hours. Since it is well documented that dramatic changes in gene expression occur within seconds in response to changes in gravity [26–29], we reasoned that the hypergravity experienced during centrifugation as well as the 4-hour incubation period with target cells under conditions of normal gravity likely prevented a truly accurate assessment of the impact microgravity would have on NK function in “real-time” during spaceflight.

Therefore, we next performed studies using a modified workflow that enabled us to maintain both the NK cells and the target tumor cells in a continuous state of microgravity throughout the entire assay period, to more accurately evaluate NK cell tumor surveillance and elimination during spaceflight and generate data that are essential for more precise astronaut cancer risk estimates. Results from the cytotoxicity assay performed in continuous sμG are shown in **Fig. 3**. As can be seen in this Figure, subjecting primary human NK cells to conditions of sμG for 48 hours and then performing the entire cytotoxicity assay under continuous conditions of sμG significantly ($p \leq 0.01$) reduced their cytotoxicity towards both K562 and MOLT-4 leukemic cell lines. Furthermore, the cytotoxicity of primary NK cells towards both leukemic cell lines was nearly identical whether the NK cell were cultured under normal gravity in standard T-flasks (1G Flask) or in stationary horizontal HARVs (1G HARV), suggesting the culture vessel’s material and geometry had no appreciable effect on NK cytotoxicity.

Continuous sμG Further Impairs the Cytotoxicity of Primary Human NK Cells beyond that of 48-hour Culture in sμG Alone

As shown in **Fig. 4**, when the results of the two different methodologies we employed (traditional cytotoxicity assay vs maintaining sμG throughout the entire assay procedure) regarding the impact sμG exerts on human NK cytotoxicity towards T-ALL (MOLT-4) are directly compared, it is readily apparent that maintaining sμG throughout the entire assay procedure leads to a statistically significant ($p \leq 0.05$) further reduction in cytotoxicity relative to the traditional assay. This difference suggests that previous reports may actually have underestimated the true reduction in NK activity caused by microgravity. Importantly, our findings also indicate that NK cells at least partially regain cytotoxic function over a period of as little as 4 hours when returned to normal gravity. When coupled with our prior report of human T-ALL in mice whose hematopoietic system was repopulated with human hematopoietic stem cells (HSC) exposed to Mars mission-equivalent doses of GCR ions [10], the marked reduction in cytotoxicity of NK cells against T-ALL when maintained in conditions of sμG raises the troubling possibility that the combined exposure to space radiation and conditions of microgravity may place astronauts at even greater risk of this malignancy during prolonged missions beyond LEO.

Interestingly, the cytotoxicity towards K562 cells did not differ significantly between the two assays (*data not shown*). The reasons for are not clear but could be a result of K562 cells exhibiting reduced expression of HLA class I and heightened expression of multiple ligands for activating NK receptors, which makes them exceptionally susceptible to NK cell-mediated cytotoxicity [44,45]. As such, even the continuous presence of conditions of sμG throughout the entire assay procedure is not able to further abrogate the ability of NK cells to recognize and kill this optimized target cell line beyond the roughly 65% inhibition resulting from the 48 hours of pre-incubation in conditions of sμG.

## RNA-seq to Delineate Mechanism(s) Whereby sμG Reduces NK Cytotoxicity

To gain insight into the mechanisms by which exposure to sμG reduces the ability of primary human NK cells to recognize and lyse hematological tumors, we performed RNA-seq analysis on RNA isolated from the NK cells of 2 healthy human donors following 48 hours of culture under conditions of sμG. As can be seen in **Fig. 5**, the NK cells from the two different donors clustered together as a function of whether they were cultured in normal gravity (1G) or sμG by both PCA (Fig. 5A) and heatmap (Fig. 5B), and the expression levels of 2184 genes were significantly ($p < 0.05$) up- or down-regulated by ≥ 1.5-fold as a result of culture in sμG, as shown in the Volcano plot in **Fig. 5C**.

Looking at specific examples of genes whose transcripts were differentially regulated by exposure to sμG, expression of various CD1 isoforms has been shown to inhibit NK cell-mediated killing [46,47]. In these prior studies, the CD1 isoforms to be tested were expressed on the target cells; however, our data show (Fig. 6) that culture in sμG leads to a marked induction of mRNA for CD1b, CD1c, CD1d, and CD1e in the NK cells themselves ($p = 2.21e^{-3}$, $8.43e^{-3}$, $7.79e^{-3}$, *and* $1.87e^{-3}$, respectively). Exposure to sμG also led to a significant up-regulation of the following transcripts (Fig. 7): 1) CD3D ($p = 1.14e^{-3}$), which is consistent with the recently described NK2.4 subset of memory-like NK cells that accumulates with aging and exhibits low expression of the TCR complex [48]; 2) CD5 ($p = 6.6e^{-3}$), a cell surface protein that has an inhibitory function in antigen receptor signaling and is not normally expressed on NK cells and would be expected to impair NK functionality [49–51]; 3) decay-accelerating factor/CD55 ($p = 8.53e^{-3}$), a molecule whose expression has been shown to inhibit human NK cell cytotoxicity against hematological cancers [52]; 4) CD84 ($p = 0.01$), engagement of which significantly dampens NK cell cytotoxicity [53]; 5) CD200 ($p = 0.02$), the shedding of which in the tumor microenvironment leads to NK cell dysfunction and apoptosis [54]; 6) CD47 ($p = 0.05$), a finding of particular relevance to the anti-tumor function of NK cells, since prior studies have shown that CD47 may play an inhibitory role in NK cell-mediated cytotoxicity against cancer cells [55]; 7) LAIR1 ($p = 0.01$), a potent inhibitor of NK cytotoxicity [56]; 8) NFATC1 ($p = 0.02$), a negative regulator of NK cell tumor immunosurveillance [57]; and 9) CD34 ($p = 7.6e^{-3}$), an antigen only present on the earliest precursors of the NK lineage [58], suggesting that microgravity may provoke a regression of NK cells to an immature, undifferentiated stem-like state, as has been reported with other cell types grown in microgravity [59,60].

Conversely, exposure to sμG led to a significant down-regulation of the following transcripts (Fig. 8): 1) KLRC3/NKG2E ($p = 0.01$), a *de facto* NK activating receptor [61]; 2) NOTCH2NLR ($p = 6e^{-3}$), a receptor in a signaling pathway that plays a substantial role in human NK cell maturation and tumor killing [62]; and 3) TNFRSF10C (p = 0.03), a molecule that is thought to protect cells from TRAIL-induced apoptosis [63]. Collectively, the differential expression of myriad genes that are intimately involved in NK cytotoxicity/function provides multiple viable mechanistic explanations for the marked reduction we observed in NK killing under conditions of sμG. Interestingly, exposure to conditions of sμG also led to marked ($p = 0.05$) induction of CD8A expression in NK cells (Fig. 9). Importantly, CD8 + NK cells have been shown to suppress CD4 + T cell activation and proliferation [64], suggesting that microgravity may also indirectly impact the function of the acquired immune system via its effects on NK cells.

## CONCLUSIONS

Taken together, these data demonstrate that even fairly brief exposure to conditions of simulated microgravity (sμG) markedly reduces the cytotoxicity of both the NK-92MI cell lines and primary human NK cells from 3 different donors towards two different hematological tumor cell lines (K562 and MOLT-4). Furthermore, these studies are the first report in which a cytotoxicity assay was conducted in conditions of continuous sμG, preventing any alterations to cells which may be caused by return to normal gravity or the hypergravity during centrifugation that is performed during standard cytotoxicity assays. While never addressed before in this setting, we reasoned that maintaining sμG throughout the duration of the assay is critical to accurately assess the true impact microgravity exerts on NK cell function, as even a few seconds of exposure to altered gravitational forces can dramatically affect gene expression [26–29]. Our observation of the further reduction in NK cell killing of MOLT-4 (T-ALL) cells when cytotoxicity assays are performed under continuous sμG vs. the standard protocol validated our prediction and demonstrated that prior studies employing traditional assay protocols [17–25] likely underestimated the impact of microgravity on NK function. The fact that a significant further decrease in killing was not observed in continuous sμG when K562 cells were used as targets raises the possibility that the exclusive use of this optimized target cell line with its exquisite sensitivity to NK lysis has the potential to mask important yet subtle effects various stressors may exert on NK function and highlights the importance of including additional target cell lines in future studies. When combined with our prior report of human T-ALL in mice repopulated with human HSC that had been exposed to Mars mission-equivalent doses of GCR ions [10], the further reduction in cytotoxicity of primary human NK cells against T-ALL when maintained in conditions of sμG raises the troubling possibility that the combined exposure to space radiation and conditions of microgravity may have the potential to increase astronaut risk of this hematological malignancy, and perhaps others, during prolonged missions beyond LEO. Importantly from the standpoint of astronaut safety, RNA-seq analysis performed on primary NK cells from two different healthy human donors revealed that multiple pathways that are central to NK function are altered by exposure to sμG, paving the way for future studies to test agents designed to target these specific pathways to serve as much-needed medical countermeasures that can mitigate the microgravity-induced deficits in NK function, enabling astronaut immune systems to effectively recognize and eliminate any malignant clones that arise as a result mutations induced by exposure to space radiation.

## Declarations

#### Acknowledgements:

This work was supported by Grant NNX17AE49G from NASA Space Biology and by the NASA Translational Research Institute for Space Health (TRISH) through Cooperative Agreement NNX16AO69A. B.M.K. and J.H.D. are supported by T32 pre-doctoral fellow positions through NIH NIBIB 2T32EB014836.

#### Author Contributions:

B.M.K and J.H.D. executed experiments, performed data analysis and interpretation; B.M.K developed method for performing NK cytotoxicity assay under continuous microgravity; T.S., K.D.R., and S.J.W. performed RNA-seq experiments and analyzed resultant data; A.A. provided reagents, resources, and experimental feedback; B.M.K. drafted manuscript; C.D.P. and G.A.P. conception and experimental design, supervised experiments, performed data analysis and interpretation, wrote final version of the manuscript, performed analysis of RNA-seq data using Rosalind, and secured funding.

#### Data Availability:

The main data supporting the results of this study are available within the paper. Source data are provided with this paper. Next generation sequencing data generated in this study have been deposited into GEO Series Accession Number 255750.

#### Competing interests:

All authors declare no financial or non-financial competing interests.

## Figures

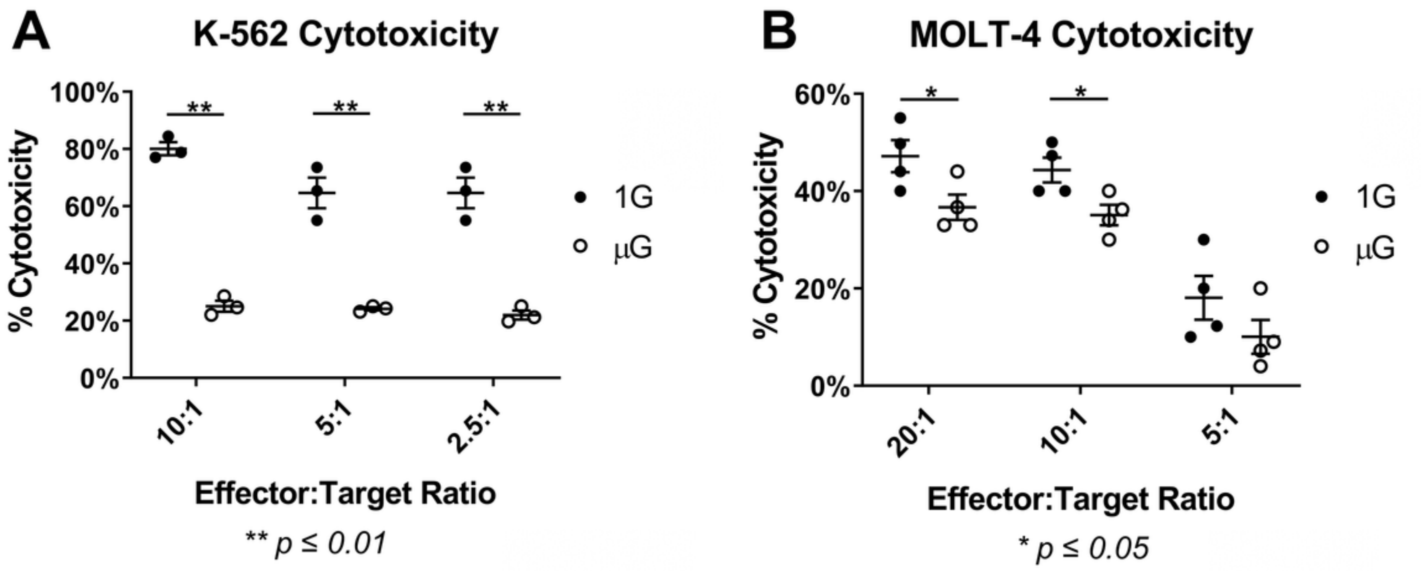

Figure 1

**Impact of simulated microgravity (smG) on NK-92MI cytotoxicity.** NK-92MI cells were exposed to smG in a rotating HARV for 48 hours and then plated with leukemic cell lines are various concentrations and incubated in normal gravity for 4 hours. Cytotoxicity against both cell lines was reduced at all E:T ratios. *n = 3 or 4*.

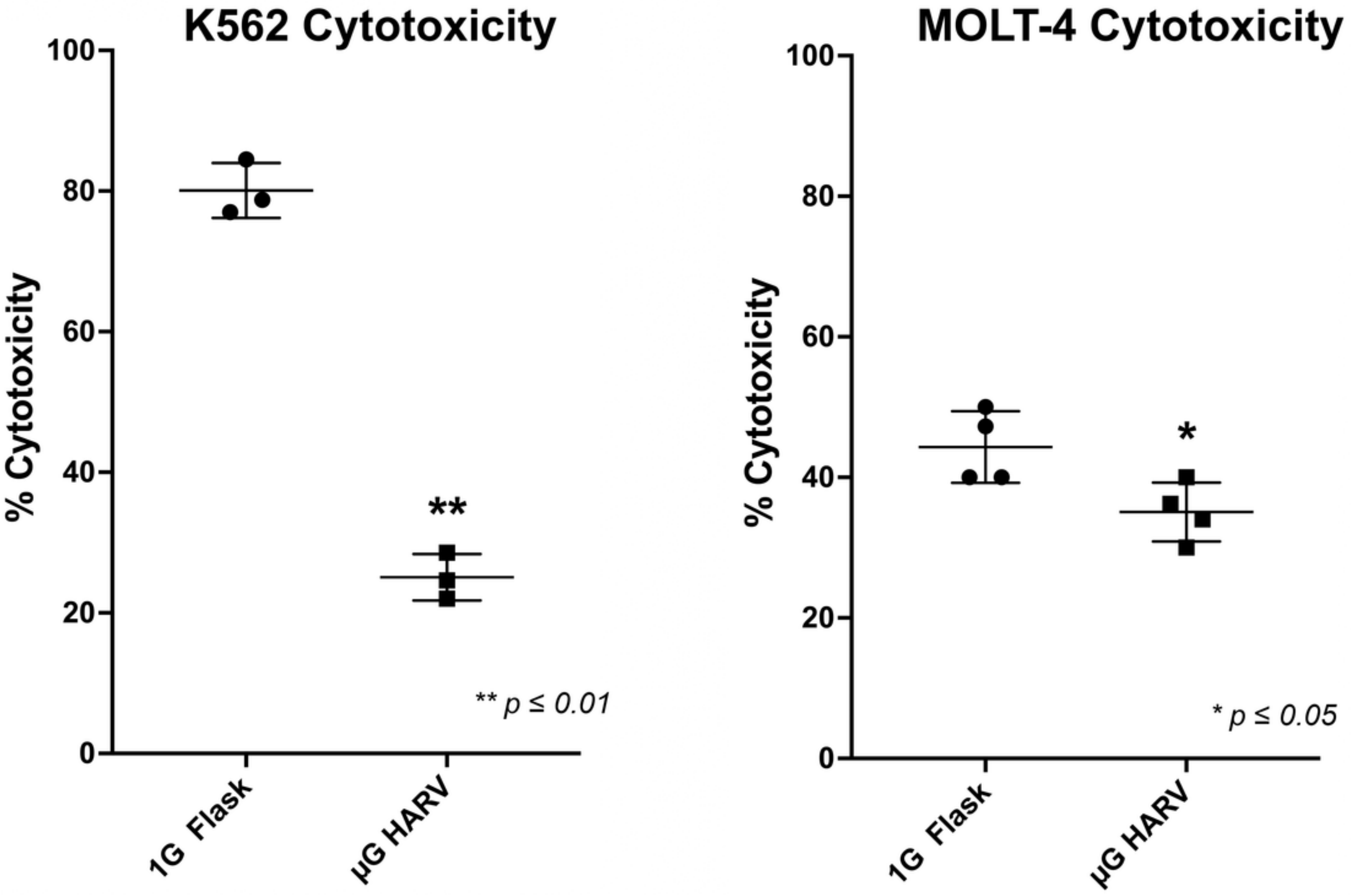

Figure 2

**Impact of smG on cytotoxicity of primary human NK cells.** Primary human NK cells were isolated from three healthy adult donors and exposed for 48 hours to: 1) normal gravity (1G) in a standard flask; 2) normal gravity (1G) in a stationary horizontal HARV; or 3) smG in a rotating HARV. NK cells were then harvested, plated with K562 and MOLT-4 leukemic cell lines at a 10:1 ratio, and incubated in normal gravity for 4 hours. Cytotoxicity against both leukemic cell lines was significantly reduced by a 48 hour exposure to smG. No significant differences were observed between tissue flasks and HARVs under normal gravity. *n = 3 separate healthy donors*.

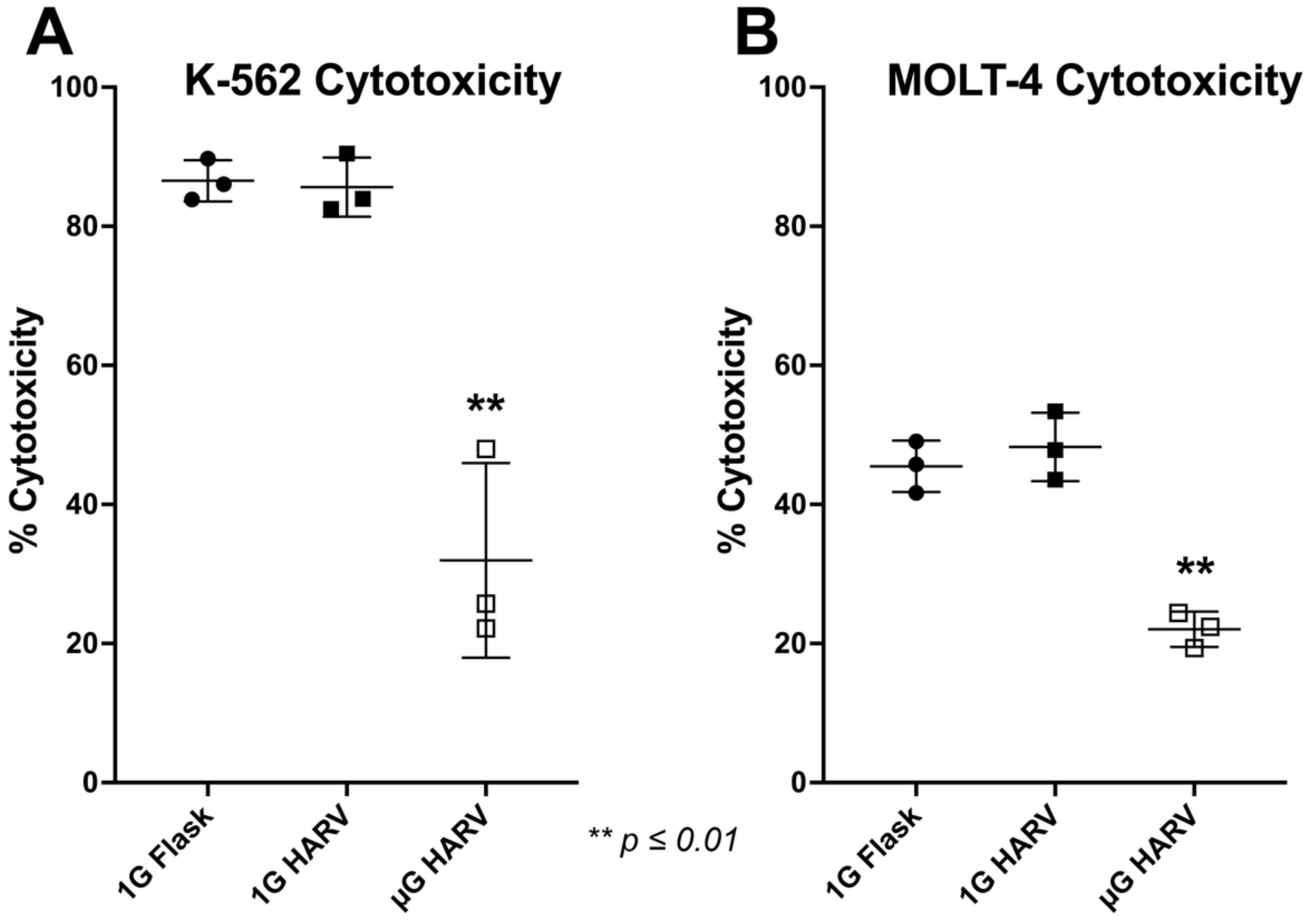

Figure 3

**Impact of continuous smG during cytotoxicity assay on NK killing.** Primary human NK cells were isolated from three healthy adult donors and exposed for 48 hours to: 1) normal gravity (1G) in a standard flask; 2) normal gravity (1G) in a stationary horizontal HARV; or 3) smG in a rotating HARV. K562 or MOLT-4 leukemic cell lines were then added at a 10:1 ratio to the vessel in which the NK cells were growing and incubated for an additional 4 hours, either under 1G (flask and horizontal stationary HARV) or under smG (rotating HARV). Primary NK cell cytotoxicity against both leukemic cell lines was significantly reduced when the cytotoxicity assay was performed under conditions of continuous smG. *n = 3 separate healthy donors*.

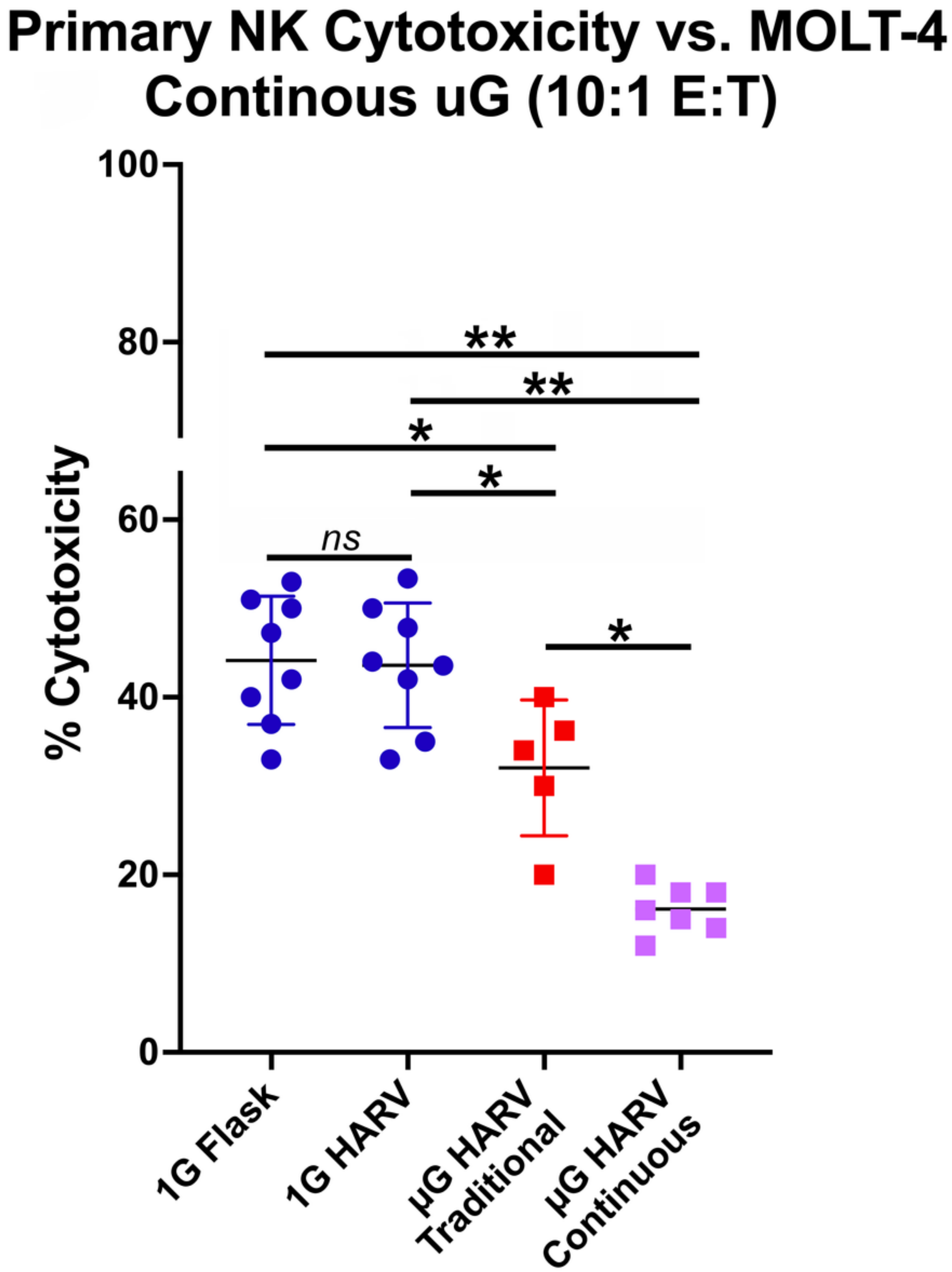

Figure 4

**Impact on primary NK cell cytotoxicity of continuous smG during cytotoxicity assay vs only a 48 hour exposure to smG.** Primary human NK cells were isolated from three healthy adult donors and exposed for 48 hours to: 1) normal gravity (1G) in a standard flask; 2) normal gravity (1G) in a stationary horizontal HARV; or 3) smG in a rotating HARV. Cytotoxicity against the K562 and MOLT-4 leukemic cell lines (at a 10:1) ratio was then assessed under standard 1G conditions or under conditions of continued smG to enable comparison of the impact preexposure vs continuous exposure to smG had on primary human NK cells function. Primary NK cell cytotoxicity against both leukemic cell lines was significantly further reduced when NK cells were pre-exposed for 48 hours to smG and the cytotoxicity assay was performed under conditions of continuous smG compared to 48 hour pre-exposure alone. *n = 3 separate healthy donors*.

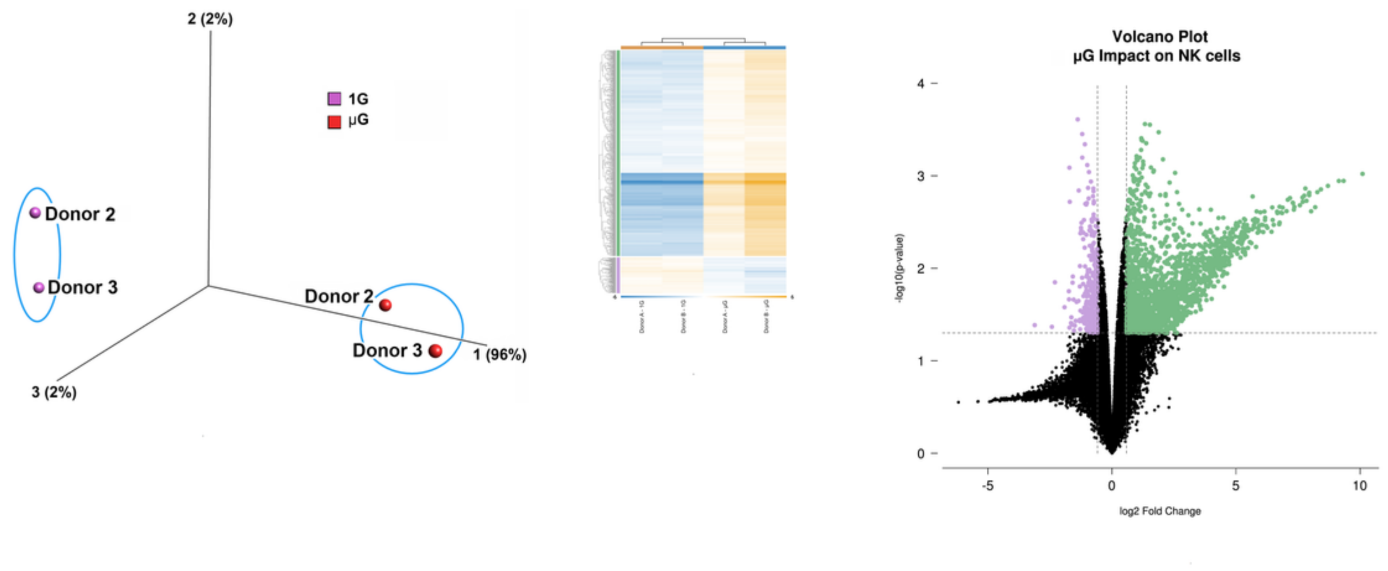

### Figure 5

**RNA-seq to identify mechanisms whereby microgravity impairs NK function.** Primary human NK cells were isolated from two healthy adult donors and exposed for 48 hours to: 1) normal gravity (1G) in a stationary horizontal HARV; or 2) smG in a rotating HARV. RNA was then isolated and subjected to RNA-seq to identify differentially expressed genes (DEG) between these two gravitational conditions. **A)** Principal Component Analysis (PCA) plot of the transcriptome of the NK cells from the two donors when cultured under smG or 1G. **B)** Heatmap plot of the transcriptome of the NK cells from the two donors when cultured under smG or 1G. **C)** Volcano plot of the transcriptome of the NK cells from the two donors when cultured under smG or 1G.

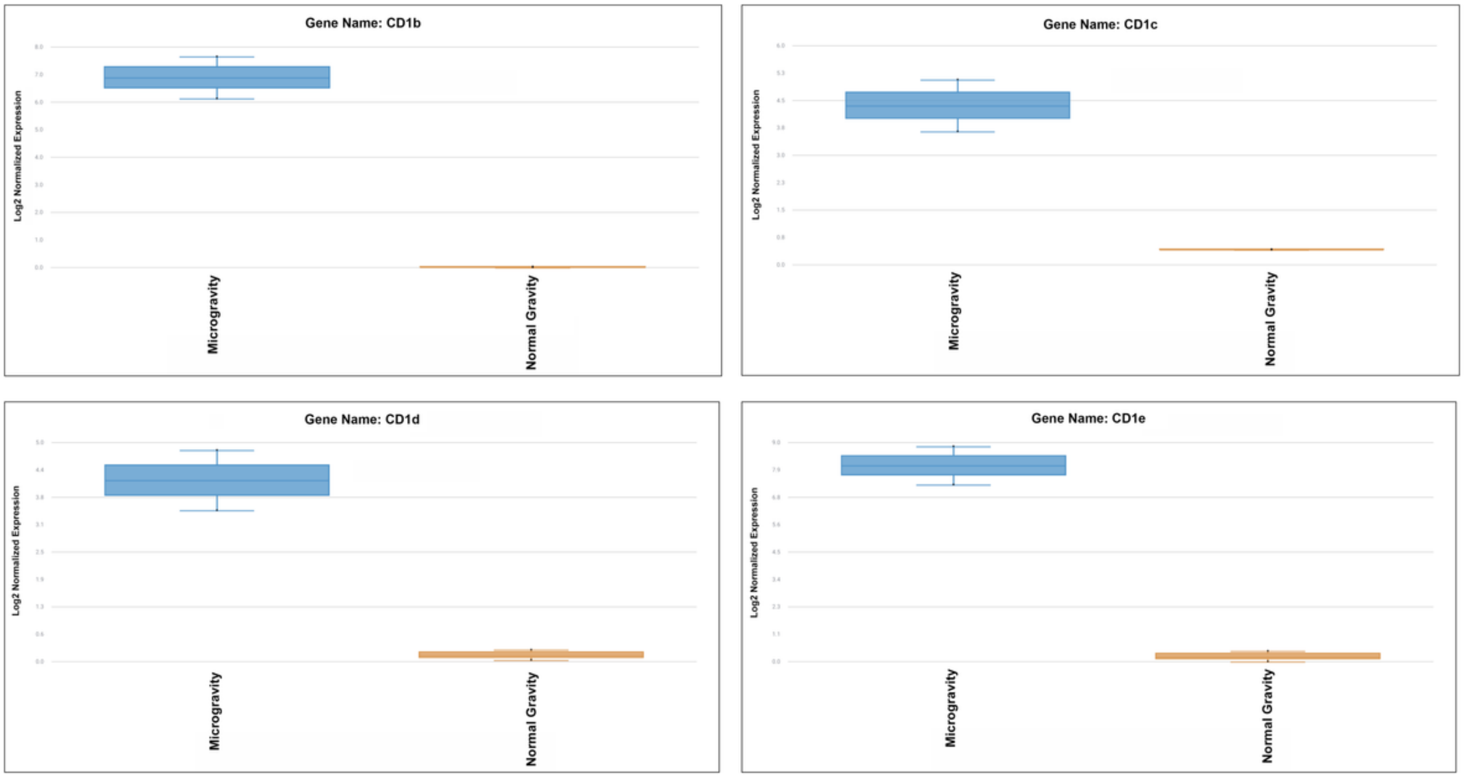

### Figure 6

**Exposure to smG leads to a marked induction of mRNA for multiple isoforms of CD1 in primary human NK cells.** Box plots of the relative expressions levels of the mRNA for CD1b, CD1c, CD1d, and CD1e in primary NK cells from two different healthy adult donors cultured under smG or 1G. Error bars represent the minimum and maximum observed values, and the line at the center of each box represents the median value ($p = 2.21e^{-3}$, $8.43e^{-3}$, $7.79e^{-3}$, *and* $1.87e^{-3}$ for CD1b, CD1c, CD1d, and CD1e, respectively).

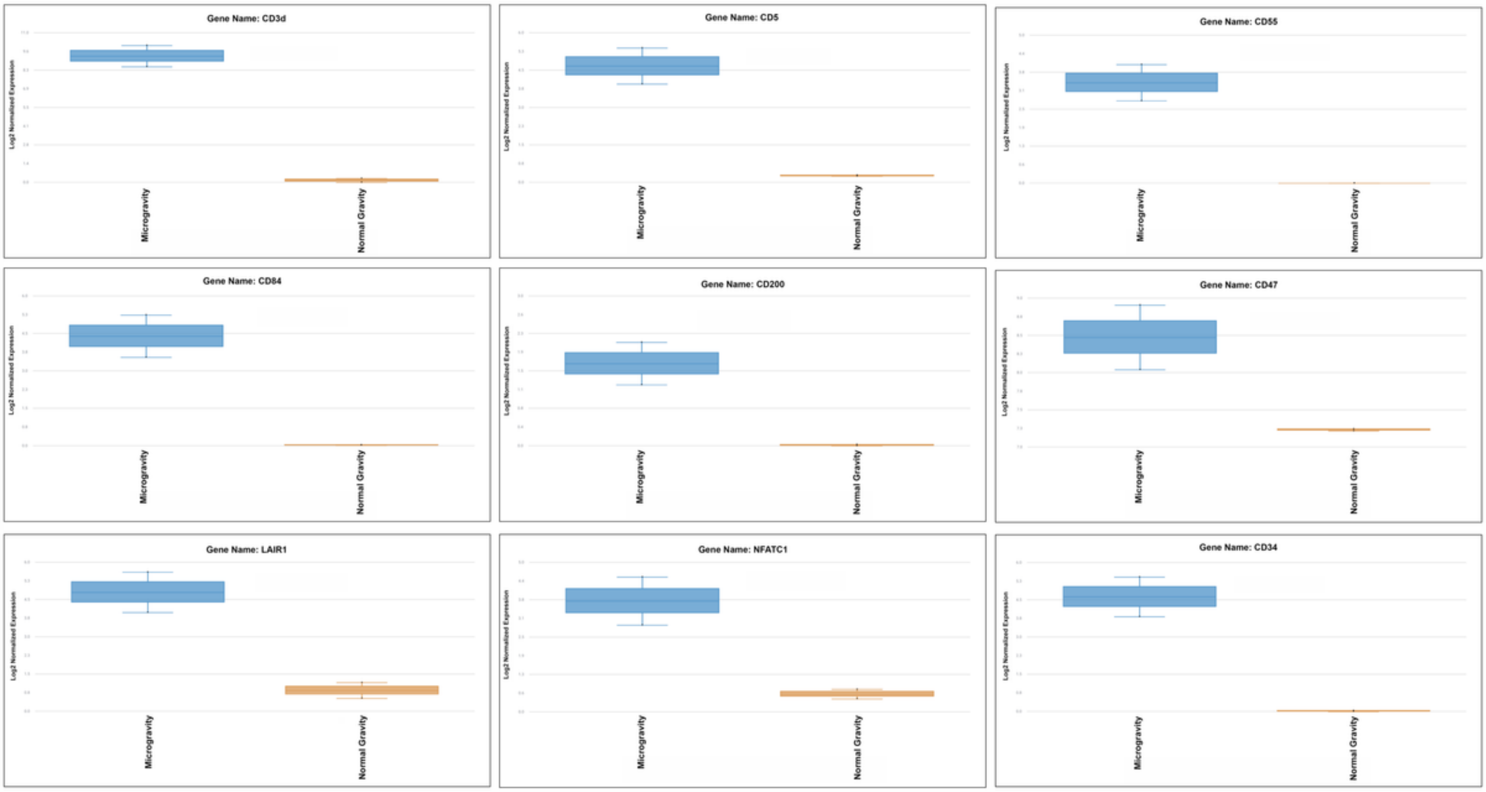

### Figure 7

**Culture under smG induces expression of multiple transcripts that may explain decreased NK cytotoxicity.** Box plots of the relative expressions levels of selected mRNAs that are up-regulated in primary NK cells from two different healthy adult donors cultured under smG vs. 1G. Error bars represent the minimum and maximum observed values, and the line at the center of each box represents the median value ($p = 1.14e^{-3}$, $6.6e^{-3}$, $8.53e^{-3}$, *0.01, 0.02, 0.05, 0.01, 0.02, and* $7.6e^{-3}$ for CD3D, CD5, CD55, CD84, CD200, CD47, LAIR1, NFATC1, and CD34, respectively).

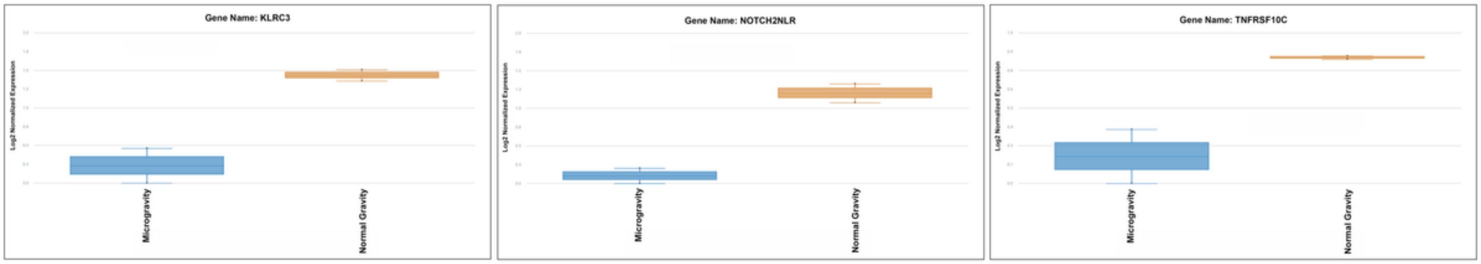

### Figure 8

**Culture under smG decreases expression of transcripts that may explain decreased NK cytotoxicity.** Box plots of the relative expressions levels of selected mRNAs that are down-regulated in primary NK cells from two different healthy adult donors cultured under smG vs. 1G. Error bars represent the minimum and maximum observed values, and the line at the center of each box represents the median value ($p = 0.01$, $6e^{-3}$, *and 0.03* for KLRC3/NKG2E, NOTCH2NLR, and TNFRSF10C, respectively).

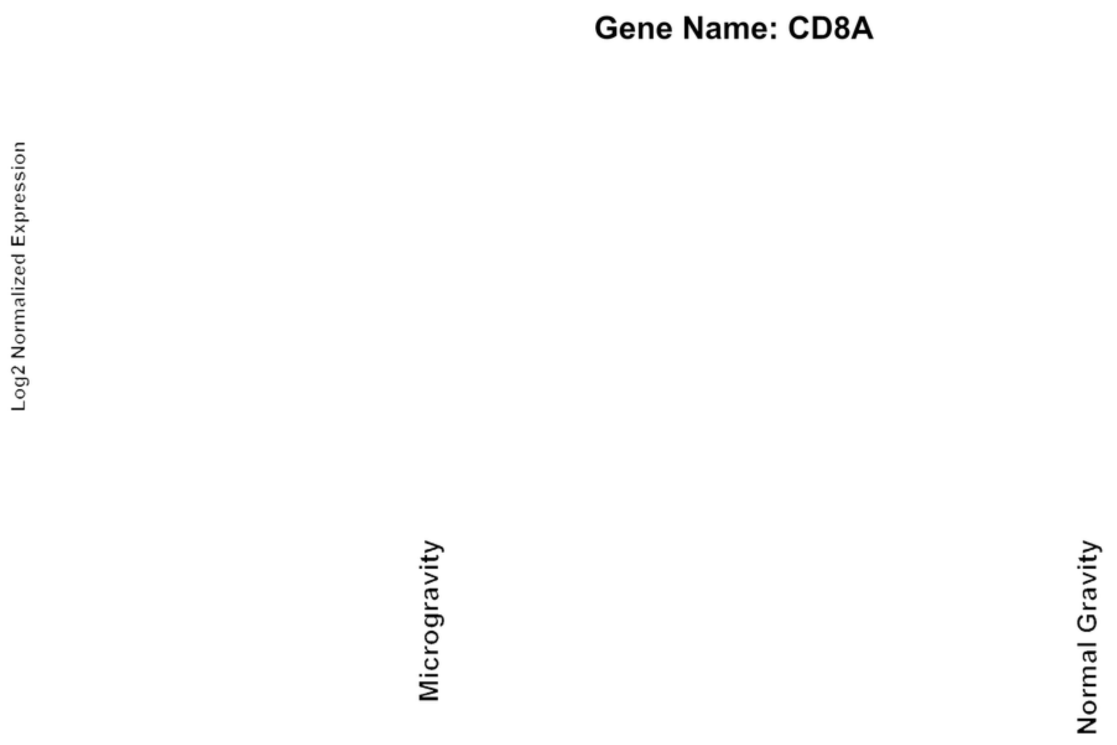

**Figure 9**

**Culture under smG induces expression of CD8A in primary human NK cells.** Box plots of the relative expressions levels of CD8A mRNA in primary NK cells from two different healthy adult donors cultured under smG vs. 1G. Error bars represent the minimum and maximum observed values, and the line at the center of each box represents the median value (*$p = 0.05$*).
